# Nurse evaluation of stress levels during CPR training with heart rate variability using smartwatches according to their personality: A prospective, observational study

**DOI:** 10.1371/journal.pone.0268928

**Published:** 2022-06-08

**Authors:** Hye Ji Park, Daun Choi, Hang A. Park, Choung Ah Lee

**Affiliations:** 1 Department of Emergency Medicine, Hallym University, Dongtan Sacred Heart Hospital, Hwaseong-si, Gyeonggi-do, Republic of Korea; 2 Hallym Dongtan Simulation Center, Hwaseong-si, Gyeonggi-do, Republic of Korea; The University of Mississippi Medical Center, UNITED STATES

## Abstract

**Background:**

Cardiopulmonary resuscitation (CPR) is a very critical phenomenon, and to prepare for it, most nurses undertake simulation training, during which learners’ stress levels should be managed. This study aims to evaluate nurses’ stress levels during CPR simulation training using heart rate variability (HRV) measured with a smartwatch and to determine the correlation between individual personality traits and stress levels.

**Methods:**

This prospective observational study was conducted from July 2020 to December 2021. For nurses participating in advanced life support training with more than six months of clinical experience, their stress levels while performing as a CPR team leader were measured. Regarding stress levels, heart rate data measured with a smartwatch were processed using Kubios HRV Standard software to generate HRV parameters. The personality of participants was evaluated using the Big Five personality test. The degree of stress according to personality was determined using HRV parameters. Consequently, the correlation between personality and stress according to the clinical experience of cardiac arrest was analyzed.

**Results:**

Of the 132 participants, 91.7% were female, and the median age of the sample was 27 years. Agreeable personality had the highest score (32.84±3.83). LF power (r = 0.18, p = 0.04) and HF power (r = 0.20, p = 0.02) showed a significant positive correlation with the agreeableness trait. In subgroup analysis according to the cardiac arrest experience, the agreeableness trait had a positive correlation with a standard deviation of NN intervals (r = 0.24, p = 0.01), root-mean-square of successive differences (r = 0.23, p = 0.02), LF Power (r = 0.26, p = 0.01), and HF power (r = 0.23, p = 0.02), but a negative correlation with mean HR (r = -0.22, p = 0.03).

**Conclusion:**

The clinical experience in cardiac arrest and agreeableness were related to acute stress during training. In the future, it is necessary to apply a scenario of a level suitable for individual personality and experience, and evaluate the level and achievement of students.

## 1. Introduction

Cardiopulmonary resuscitation (CPR) can cause significant mental stress for the rescuers, leading to attention deficit and increased distractibility [1]. This, in turn, can lead to misjudgment of priorities and delay in CPR performance, lowering the quality of CPR performance and further increasing mental stress [2]. Most nurses are undertaking simulation training to improve the effectiveness of CPR performance and reduce the burden of real situations. Simulation education not only increases knowledge but also helps medical personnel boost their knowledge in actual clinical practice, reduces anxiety among medical personnel, increases teamwork, and boosts self-efficacy [3,4].

To reveal the optimal educational effect, the learner’s cognitive load and stress level should be considered in educational design [5]. Theoretically, stressful training helps learners prepare for a high-stress clinical environment, but overly stressful training can cause emotional exhaustion, potentially producing negative effects [6]. Therefore, it is necessary to maintain an appropriate level of stress while undertaking training [7] and perform appropriate management of stress [2]. More work is required to determine how stress levels during training affect learning and eventual performance in real-world situations [8].

The effect of stress depends not only on the exact level of the stress itself but also on several other factors, such as how one perceives and evaluates a given situation, whether one can get appropriate help from others, and how rationally one responds [9]. Previous research found that the degree of stress is correlated with personality [7,10,11].

For stress management, the stress level must be assessed objectively in real-time. Several recent studies have demonstrated that heart rate variability (HRV) computed using a smartwatch is an appropriate measure of acute stress [12,13].

This study aims to evaluate the learners’ stress levels while undertaking CPR simulation training using HRV parameters measured using a smartwatch and to investigate the correlation between individual personality traits and stress.

## 2. Method

### 2.1. Study design and participants

This prospective, observational study was conducted between July 2020 and December 2021. The participants were nurses who had worked at the current hospital for more than six months, understood the research objectives, and agreed to participate in the study. Participants with arrhythmias or on medications that affect heart rate (HR) were excluded from the study.

### 2.2. Training course and simulation

The nurse students were provided with the Korean Advanced Life Support (KALS) training program by the Korea Association of Cardiopulmonary Resuscitation. The KALS program included an introduction (20 minutes), slide-based lectures (40 minutes), procedural skills training (80 minutes), scenario-based simulation training (60 minutes), a performance test (60 minutes), and a summary session (10 minutes). Simulation training consisted of one instructor and five to six trainees per group, and each trainee took turns acting as the team leader, while the rest were team members. In this study, HR was measured by adding five-minute simulations between scenario-based simulation training and a performance test. In the KALS course, four scenarios were repeatedly presented in the order shown in S1 Table.

### 2.3. Data collection and processing

When performing the role of a team leader in the CPR simulation, the participants’ HR was monitored using an Apple Watch Series 4, 5, or 6 smartwatch (Apple Inc). After the instructor presented the background situation of the scenario, the participants performed CPR on the patient, during which their HRs were measured. To increase measurement accuracy, an elastic wristband was worn on top of the smartwatch to strengthen contact with the smartwatch and to prevent the trainee from checking their HR. Raw data were obtained by recording the screen of a smartphone paired with a smartwatch, and it was measured for five minutes. Raw heart rate data were processed utilizing the Kubios HRV Standard software (Kubios Oy, Kuopio, Finland) to generate HRV parameters [14].

### 2.4. Measurement

One week before the training, a pre-questionnaire was distributed online to check age, gender, experience in CPR training and actual sighting, and personality trait. (S2 Table) For personality type classification, the Korean version of the modified version [15] of the Big Five personality traits developed by Costa and McCrae was used [16]. This scale has a total of 44 items, and each item is rated on a scale of 1 to 5. Based on the scores, students’ personality traits were identified from the following: extraversion, agreeableness, conscientiousness, neuroticism, and openness.

HRV parameters are divided into time domains and frequency domains. Time-domain indices of HRV indicate the amount of variability in measurements of the interbeat interval (NN interval), which is the time period between successive heartbeats. Frequency-domain indices are the distribution of absolute or relative power into four frequency bands. Mean HR, the standard deviation of NN intervals (SDNN), root mean square of successive differences (RMSSD), the proportion of NN50 divided by the total number of NNs (pNN50) in the time domains, and low frequency (LF, 0.04~0.15 Hz) power, high frequency (HF, 0.15~0.4 Hz) power, and LF/HF ratio in the frequency domain domains, which were proven to reflect acute stress, the primary outcome [17,18]. Further, HRV parameters according to the personality trait of the two groups were compared by dividing them into two groups according to the presence or absence of clinical experience in cardiac arrest.

### 2.5. Statistical analysis

Statistical analysis was performed using SPSS Version 25.0 (IBM Corp., Armonk, NY, USA) with statistical significance defined as p < 0.05. Continuously distributed variables were presented as means/ standard deviation (SD) or median/interquartile range (IQR), and categorical variables were presented as percentages (%). Regarding the relationship between HRV parameters and personality traits, since the HRV parameters did not have normality, a statistical method using the non-parametric Spearman’s correlation analysis was utilized. Spearman correlation analysis can be used when the sample size of two continuous variables is small and normality is not satisfied or when a ranking scale is included [19]. It is not possible to estimate a linear relationship between two variables. It indicates whether when one variable increases, the other variable also tends to increase. A coefficient ranges from –1 to +1. It can be interpreted as describing anything between no association (rho = 0) to a perfect monotonic relationship (rho = –1 or +1). A coefficient of <0.1 indicates a negligible and >0.9 indicates a very strong relationship and a coefficient of 0.1 to 0.3 indicates a weak correlation [20].

### 2.6. Ethics

This study was approved by the institutional review board of Hallym University (HDT 2020-07-003), and all participants provided informed consent.

## 3. Results

### 3.1. General characteristics of participants

**Fig 1:**
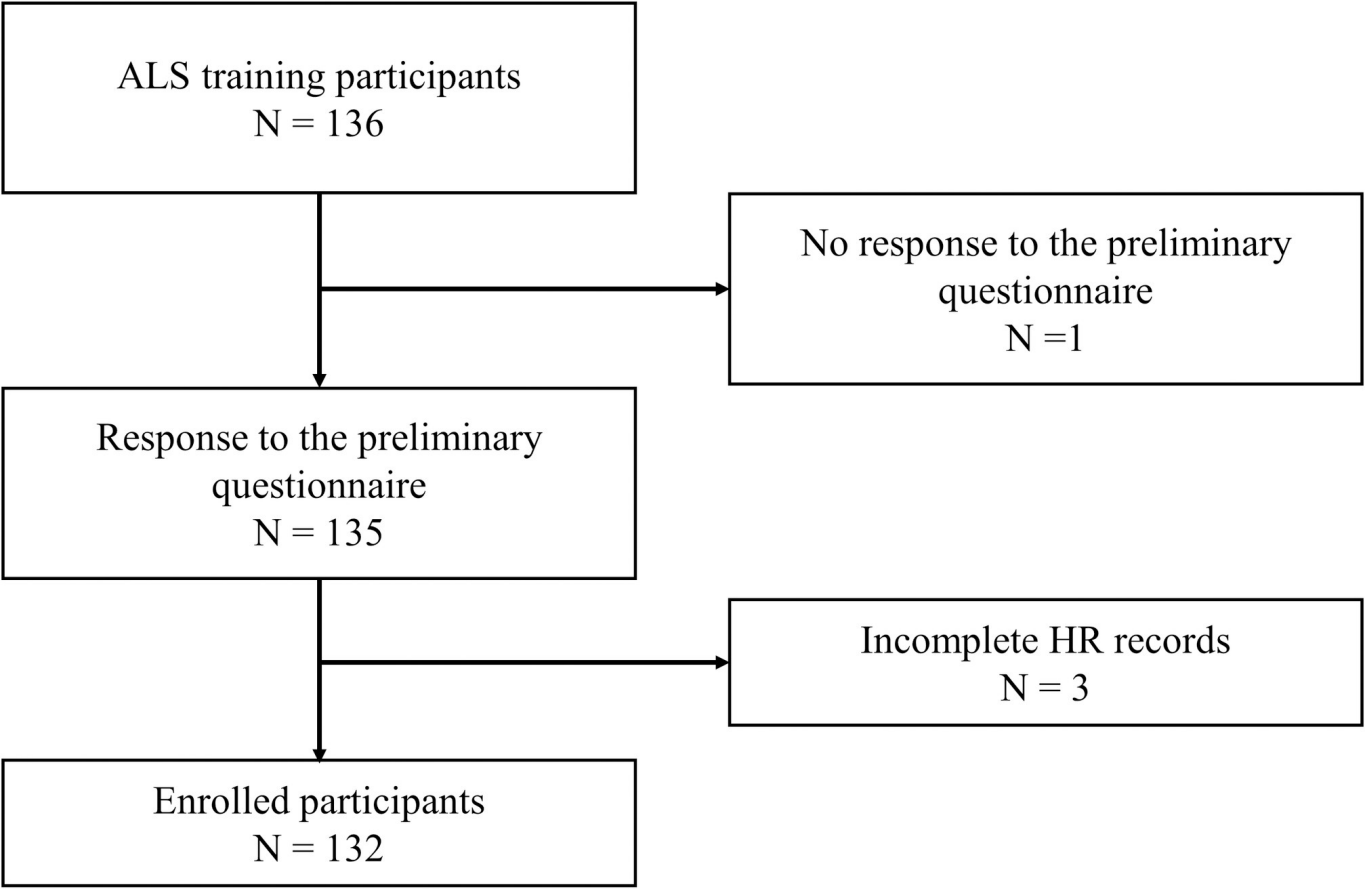
Flow diagram of participant enrollment.

Among the 136 participants, 1 did not respond to the preliminary questionnaire and 3 had incomplete HR records and, thus, were excluded from participation. Finally, 132 students were enrolled (Fig 1).

The median age was 27 years (IQR, 24.25–31.75), and 122 (91.7%) of the sample were female. Forty nine (37.1%) had received basic life support (BLS) training and 55 (41.7%) had received advanced life support (ALS) training within the two years prior to evaluation. About 76% had a clinical experience with cardiac arrest; 77 (58.3%) had participated in resuscitation. Their roles in the CPR team were airway management (13.6%), compression (37.1%), defibrillation (12.9%), and intravenous (IV) access or drug administration (Table 1).

**Table 1:** Baseline characteristics of participants.

	Total N = 132
Age, years	27 (24.25–31.75)
Sex, female	122 (91.7)
Previous BLS training < 2 years	49 (37.1)
Previous ALS training < 2 years	55 (41.7)
Clinical experience of cardiac arrest	100 (75.8)
Clinical experience as a resuscitation team member	77 (58.3)
Main role during CPR	
Airway	18 (13.6)
Compression	49 (37.1)
Defibrillation	17 (12.9)
IV access/drug administration	61 (46.2)

Data are presented as median (interquartile range) or number (%) or mean (standard deviation). BLS, basic life support; ALS, advanced life support; CPR, cardiopulmonary resuscitation; IV, Intravenous.

**Fig 2:**
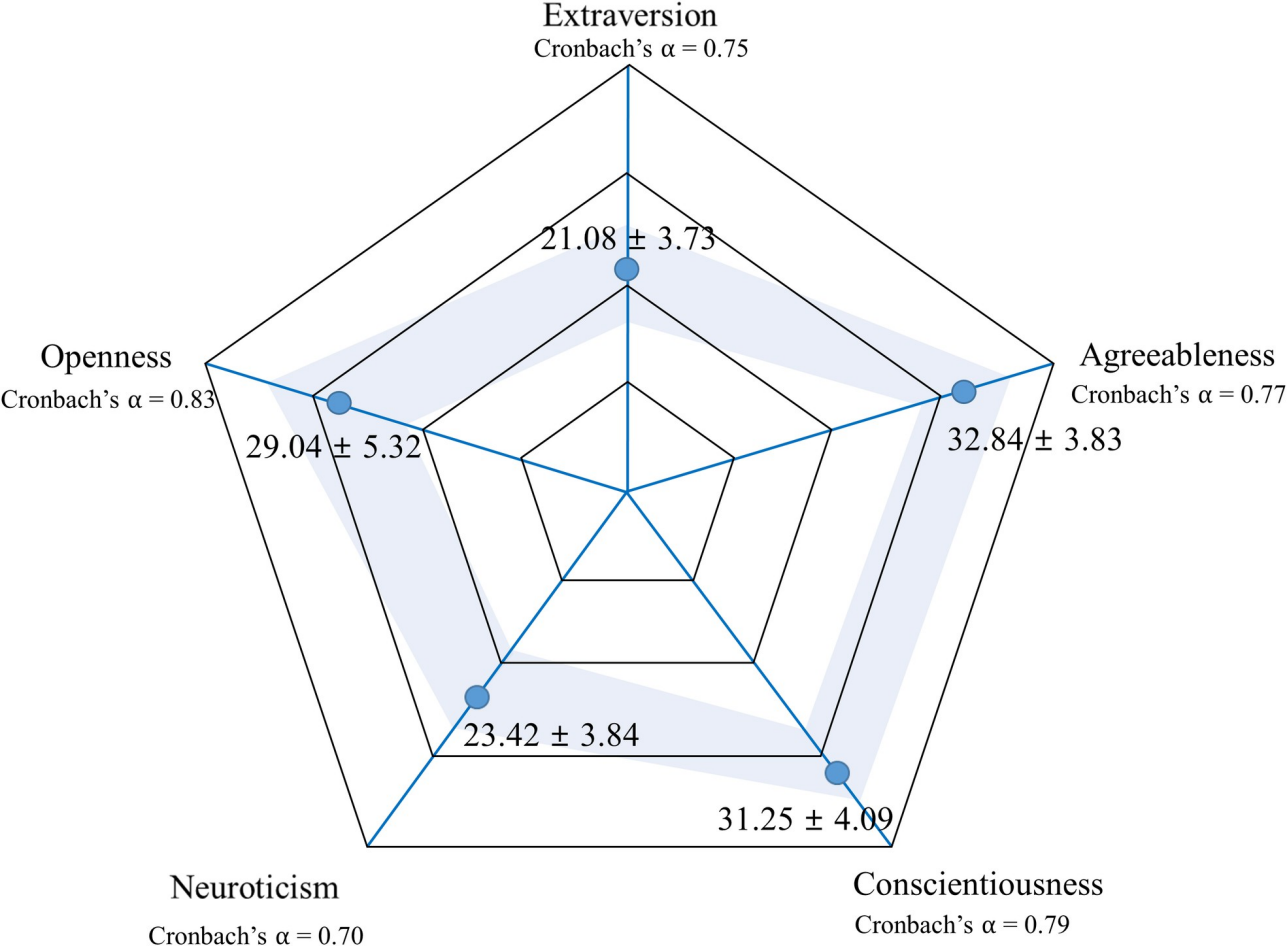
Score distribution according to the personality traits of participants. Score distribution is expressed as mean and standard deviation.

The participants’ total Cronbach’s ⍺ in the Big Five personality test was 0.69, and Cronbach’s ⍺ in the five subscales was 0.70–0.83. The scores for each personality type were in the following order: agreeableness (32.84±3.83), conscientiousness (31.25±4.09), openness (29.04±5.32), neuroticism (23.42±3.84), and extraversion (21.08±3.73) (Fig 2).

Concerning HRV parameters, mean HR is 120.55 (SD, 19.30), SDNN is 24ms (IQR, 16.85–32.35), RMSSD is 16.5ms (IQR, 11.30–23.62), pNN50 is 1.19% (IQR, 0–5.14), LF Power was 211ms2 (IQR, 90.25–500), HF Power was 120.5ms2 (IQR, 44–260), and LF/HF ratio was 2.06 (IQR, 1.02–3.49). (Table 2).

**Table 2:** HRV parameters of all participants.

	HRV values
Mean HR, /min	120.55 ± 19.3
SDNN, ms	24 (16.85–32.35)
RMSSD, ms	16.5 (11.3–23.62)
pNN50, %	1.19 (0–5.14)
LF Power, ms^2^	211 (90.25–500)
HF Power, ms^2^	120.5 (44–260)
LF/HF ratio	2.06 (1.02–3.49)

Data are shown as median (interquartile range) or mean (standard deviation).

HRV, heart rate variability, SDNN, standard deviation of all NN intervals; RMSSD, root-mean-square of successive differences; pNN50, NN50 count divided by the total number of NN intervals; LF Power, power in low-frequency range; HF power, power in high-frequency range.

HRV parameters according to personality type are shown in Table 3. LF power (r = 0.18, p = 0.04) and HF power (r = 0.20, p = 0.02) showed a significant positive correlation with agreeableness, and other personality types showed no significant correlation.

**Table 3:** Correlations between personality traits and HRV parameters.

	Mean HR	SDNN	RMSSD	pNN50	LF Power	HF Power	LF/HF ratio
Extraversion	0.01	0.13	0.11	0.02	0.14	0.12	0.05
Agreeableness	-0.16	0.15	0.15	0.01	0.18*	0.20*	-0.07
Conscientiousness	-0.08	0.05	0.01	-0.08	0.06	0.06	0.07
Neuroticism	-0.00	-0.11	-0.10	-0.00	-0.06	-0.07	-0.11
Openness	-0.10	0.08	0.04	-0.03	0.10	0.06	0.08

*p<0.05.

SDNN, standard deviation of all NN intervals; RMSSD, root-mean-square of successive differences; pNN50, NN50 count divided by the total number of NN intervals; LF power, power in low-frequency range; HF power, power in high-frequency range.

A subgroup analysis was performed on personality traits and HRV according to the individual’s clinical experience of cardiac arrest. There was no significant difference between the group with and without clinical experience, except for age and agreeable personality (p = 0.01) (Table 4).

**Table 4:** Subgroup analysis according to the cardiac arrest experience.

	Experience N = 100	No Experience N = 32	P-value
Age, years	31.0 (29.42–32.76)	25.72 (23.85–27.59)	0.01
Sex, female	92 (92.0)	30 (93.8)	0.74
Previous BLS training < 2 years	95 (95.0)	29 (90.6)	0.37
Previous ALS training < 2 years	45 (45.0)	10 (31.25)	0.17
Big Five Personality			
Extraversion	21.01 ± 3.60	21.31 ± 4.20	0.69
Agreeableness	32.37 ± 3.81	34.31 ± 3.59	0.01*
Conscientiousness	31.17 ± 4.00	31.50 ± 4.43	0.69
Neuroticism	23.49 ± 3.67	23.19 ± 4.40	0.70
Openness	29.10 ± 5.12	28.81 ± 5.95	0.79

*p<0.05.

Data are represented as median (interquartile range) or number (%) or mean (standard deviation). BLS, basic life support; ALS, advanced life support; CPR, cardiopulmonary resuscitation.

In the group with experience of cardiac arrest, the agreeableness trait has positive correlation with SDNN (r = 0.24, p = 0.01), RMSSD (r = 0.23, p = 0.02) LF Power (r = 0.26, p = 0.01), HF power (r = 0.23, p = 0.02), and a negative correlation with mean HR (r = -0.22, p = 0.03) (Table 5).

**Table 5:** Association between personality and HRV parameters according to the cardiac arrest experience.

		Mean HR		SDNN		RMSSD		pNN50		LF Power		HF Power		LF/HF ratio	
		rho	P-value	rho	p-value	rho	p-value	rho	p-value	rho	p-value	rho	p-value	rho	p-value
Extraversion															
	Experience	-0.02	0.86	0.09	0.36	0.09	0.37	0.01	0.95	0.11	0.27	0.08	0.42	0.02	0.87
	No experience	0.07	0.70	0.28	0.12	0.21	0.26	0.06	0.76	0.27	0.13	0.25	0.17	0.15	0.40
Agreeableness															
	Experience	-0.22	0.03*	0.24	0.01*	0.23	0.02*	0.09	0.37	0.26	0.01*	0.23	0.02*	-0.01	0.90
	No experience	0.09	0.64	-0.18	0.33	-0.14	0.44	-0.30	0.10	-0.13	0.47	0.03	0.89	-0.16	0.38
Conscientiousness															
	Experience	-0.15	0.15	0.01	0.93	-0.03	0.79	-0.08	0.43	0.04	0.70	-0.04	0.69	0.07	0.47
	No experience	0.10	0.58	0.14	0.46	0.06	0.76	-0.08	0.68	0.10	0.60	0.30	0.10	0.11	0.55
Neuroticism															
	Experience	0.03	0.76	-0.17	0.09	-0.13	0.19	-0.05	0.61	-0.10	0.32	-0.05	0.65	-0.17	0.09
	No experience	-0.12	0.53	0.10	0.61	0.02	0.93	0.15	0.40	0.11	0.55	-0.10	0.60	0.05	0.79
Openness															
	Experience	-0.14	0.17	0.13	0.21	0.06	0.59	-0.01	0.92	0.16	0.12	0.07	0.52	0.16	0.12
	No experience	0.04	0.82	-0.09	0.65	-0.04	0.83	-0.15	0.40	-0.10	0.57	-0.02	0.93	-0.15	0.41

*p<0.05.

SDNN, standard deviation of all NN intervals; RMSSD, root-mean-square of successive differences; pNN50, NN50 count divided by the total number of all NN intervals; LF Power, power in low-frequency range; HF Power, power in high-frequency range.

## 4. Discussion

This study is the first study to evaluate the objective stress level of CPR training according to an individual’s personality, using a smart watch. We observed a weak negative correlation between the agreeableness personality trait and stress measurements during ALS training. Since the outbreak of COVID-19, it is changing from group education to self-directed education [21,22]. Self-directed education signify that learners take charge of diagnosis learning needs, identify learning goals, select learning strategies, and evaluate learning performances and outcomes. To achieve successful self-directed education, it is necessary to provide education that reflects individual characteristics. There is a positive correlation between stress and self-regulated learning skills [23], and evidence suggests that stress may be related to an individual’s personality type [18]. The authors attempted to evaluate the correlation between an individual’s personality and stress levels.

The estimation of stress through HRV is a well-established technique [24,25]. The stress situation affects the autonomic nervous system (ANS) activity of the human body, and this activity regulates the heart’s rhythm, resulting in changes in HR through the activation of the parasympathetic nerve or the sympathetic nerve [26]. HRV reflects the dynamic balance between sympathetic and parasympathetic branches of ANS [24]. Therefore, acute stress can be measured through HRV measurement as one of the reliable biomarkers of ANS activity [25].

The most common personality traits of the participants in this study were found to be agreeableness. In the existing studies on the personality of nurses, the baselines varied from study to study. In a study that investigated clinical nurses at 10 hospitals in China, agreeableness was found to be the most common trait [27]. Neuroticism and openness traits were high in Yazdanian et al.’s study [28]. Other studies have reported that conscientiousness was the most common trait among nurses working in general wards and ICUs, and nurses having worked in a nursing position for at least one year [29].

In this study, LF and HF power showed a weak positive correlation with the agreeableness trait. This is similar to previous studies’ results that revealed that the team leader experienced less stress as the agreeableness trait was higher. It was also reported that the higher the agreeableness tendency, the higher the leadership and the higher the CPR performance [30,31].

Agreeableness refers to how people tend to treat relationships with others. Agreeableness focuses on people’s orientation and interactions with others. This personality dimension includes attributes such as trust, altruism, kindness, affection, and other prosocial behaviors. People who are high in agreeableness tend to be more cooperative [32]. Since the stress was measured when the participants were acting as the CPR team leader, it was believed that the agreeableness trait affected the stress. Conversely, people with low agreeableness can be critical, uncooperative, or suspicious [32]. For these reasons, training focused on teamwork before leader training may be beneficial.

When participants were divided into two groups—with and without experience of cardiac arrest,—there were more indicators showing a correlation with stress in the group that witnessed cardiac arrest. In the group with experience of cardiac arrest, SDNN, RMSSD, LF power, and HF power were positively correlated, and the average HR was negatively correlated, indicating that the stronger the affinity trend, the lower the stress. It is known that work experience has a significant influence on the acquisition of nursing competencies [33]. Clinical experience of cardiac arrest situations can induce successful ALS training [34] and is thought to alleviate the stress of training.

The results of this study suggest that stress during education may be partially affected by clinical experience and personality differences at the individual level. The spread of infectious diseases is accelerating personalized learning, but CPR training is conducted in the form of team-based group training. In the future, it should be possible to provide a level of educational difficulty that is appropriate and tailored to the individual. Additionally, the level of stress and the educational effect should be evaluated according to the educational difficulty.

There are some limitations to this study. First, there are other factors than stress that affect HR. Generally, while HRV measures resting state, we measured HRV during training. However, HRV measurements via ambulatory monitors have become common, and the accuracy is also maintained well in the moving state [35]. Further, since it is the duty of the leader to grasp and direct the situation in the field, physical movement is minimal. Moreover, an elastic wristband was additionally used to reduce noise during HRV measurement and improve smartwatch-wearing stability. Additionally, to minimize the effect of stress on educational outcomes, stress was measured before the evaluation.

Second, there is an issue with the accuracy of measurement. The heart sensor supports a range of 30–210 beats per minute. There was no extreme bradycardia or extreme tachycardia in the study participants.

Third, the level of difficulty between scenarios applied to the simulation should be maintained at the same level. Difficult tasks cause more stress. The scenarios used for the test are part of the training program that was launched in 2011 and standardized in difficulty by a group of experts.

Lastly, the sample size might have been too small to confirm the correlation with the personality traits. Therefore, the results should be verified in a larger scale study.

## Conclusion

Training is more effective when individual perception, opinions, and experiences are considered within the stress level that individuals can accept. The clinical experience with cardiac arrest and agreeable personality are related to acute stress during the CPR training. Future studies should use a larger sample to clarify personality and relevance. A scenario of a level suitable for individual personality and experience should be developed, applied, and evaluated. This could serve as the basis for personalized training to improve learning from CPR simulations.

## Supporting information

S1 Table Scenarios and ECG monitor rhythm for each evaluation case. (DOCX)

S2 Table Preliminary questionnaire. (DOCX)

S1 Data Data report. (XLSX)
