# Supplementary material for: Nurse evaluation of stress levels during CPR training with heart rate variability using smartwatches according to their personality: A prospective, observational study
Source: PLoS One. 2022 Jun 8;17(6):e0268928. doi: 10.1371/journal.pone.0268928 (PMC9176775; doi:10.1371/journal.pone.0268928)
Supplement: S1 Table — (DOCX) [file pone.0268928.s001.docx]

**S1 Table. Scenarios and ECG monitor rhythm for each evaluation case.**

| No | Scenario | ECG monitored |
| --- | --- | --- |
| 1 | General ward  71-year-old female  Medical history: Ischemic stroke, 3 years ago  She was hospitalized after surgery for a femoral neck fracture two weeks ago. A guardian suddenly asked for help because the patient was exhibiting abnormality. | VF – VF – PEA – PEA – ROSC |
| 2 | Emergency room  60-years-old male  He complained of chest pain and showed seizure-like movements as soon as he was laid on the bed. | VF – VF – VF – PEA – ROSC |
| 3 | Hospital toilet  50’s male  Security personnel found the patient and called for help. The person's face and clothing were covered with large amounts of blood and vomit. | Asystole – Asystole – PEA – VF – ROSC |
| 4 | Emergency room  45-year-old male  Medical history: Diabetes, hypertension, end-stage renal disease on hemodialysis  He presented with general weakness, nausea, and vomiting. He suddenly lost consciousness during the interview. | PEA – PEA – VF – VF– ROSC |

ECG, electrocardiogram; VF, ventricular fibrillation; PEA, pulseless electrical activity; ROSC, return of spontaneous circulation
