# Supplementary material for: Nurse evaluation of stress levels during CPR training with heart rate variability using smartwatches according to their personality: A prospective, observational study
Source: PLoS One. 2022 Jun 8;17(6):e0268928. doi: 10.1371/journal.pone.0268928 (PMC9176775; doi:10.1371/journal.pone.0268928)
Supplement: S2 Table — (DOCX) [file pone.0268928.s002.docx]

**S2 Table. Preliminary questionnaire.**

| Sex | | M / F | | | Age (year) | | | |  | | | | | |
| --- | --- | --- | --- | --- | --- | --- | --- | --- | --- | --- | --- | --- | --- | --- |
| 1. The following questions are about your education history. | | | | | | | | | | | | | | |
| Have you received basic life support training in the last 2 years? | | | | | | | | | | Y | | | N | |
| Have you received advanced life support training in the last 2 years? | | | | | | | | | | Y | | | N | |
| 2. The following questions are about the clinical experience of cardiac arrest. | | | | | | | | | | | | | | |
| Have you ever witnessed cardiac arrest while performing clinical work? | | | | | | | | | | Y | | | N | |
| Have you ever participated as a team member in a cardiac arrest situation? | | | | | | | | | | Y | | | N | |
| If yes, what role did you play? Please select all that apply | | | | | | | | | | | | | | |
|  | □ Airway | | □ Compression | | | | □ Defibrillation | | | | | □ IV access/drug administration | | |
| 3. The Big Five Inventory  Here are a number of characteristics that may or may not apply to you. For example, do you agree that you are someone who likes to spend time with others? Please write a number next to each statement to indicate the extent to which you agree or disagree with that statement. | | | | | | | | | | | | | | |
| I see myself as someone who… | | | | Disagree strongly | | Disagree a little | | Neither agree nor disagree | | | Agree a little | | | Agree strongly |
| Is talkative | | | |  | |  | |  | | |  | | |  |
| Tends to find fault with others | | | |  | |  | |  | | |  | | |  |
| Does a thorough job | | | |  | |  | |  | | |  | | |  |
| Is depressed, blue | | | |  | |  | |  | | |  | | |  |
| Is original, comes up with new ideas | | | |  | |  | |  | | |  | | |  |
| Is reserved | | | |  | |  | |  | | |  | | |  |
| Is helpful and unselfish with others | | | |  | |  | |  | | |  | | |  |
| Can be somewhat careless | | | |  | |  | |  | | |  | | |  |
| Is relaxed, handles stress well | | | |  | |  | |  | | |  | | |  |
| Is curious about many different things | | | |  | |  | |  | | |  | | |  |
| Is full of energy | | | |  | |  | |  | | |  | | |  |
| Starts quarrels with others | | | |  | |  | |  | | |  | | |  |
| Is a reliable worker | | | |  | |  | |  | | |  | | |  |
| Can be tense | | | |  | |  | |  | | |  | | |  |
| Is ingenious, a deep thinker | | | |  | |  | |  | | |  | | |  |
| Generates a lot of enthusiasm | | | |  | |  | |  | | |  | | |  |
| Has a forgiving nature | | | |  | |  | |  | | |  | | |  |
| Tends to be disorganized | | | |  | |  | |  | | |  | | |  |
| Worries a lot | | | |  | |  | |  | | |  | | |  |
| Has an active imagination | | | |  | |  | |  | | |  | | |  |
| Tends to be quiet | | | |  | |  | |  | | |  | | |  |
| Is generally trusting | | | |  | |  | |  | | |  | | |  |
| Tends to be lazy | | | |  | |  | |  | | |  | | |  |
| Is emotionally stable, not easily upset | | | |  | |  | |  | | |  | | |  |
| Is inventive | | | |  | |  | |  | | |  | | |  |
| Has an assertive personality | | | |  | |  | |  | | |  | | |  |
| Can be cold and aloof | | | |  | |  | |  | | |  | | |  |
| Perseveres until the task is finished | | | |  | |  | |  | | |  | | |  |
| Can be moody | | | |  | |  | |  | | |  | | |  |
| Values artistic, aesthetic experiences | | | |  | |  | |  | | |  | | |  |
| Is sometimes shy, inhibited | | | |  | |  | |  | | |  | | |  |
| Is considerate and kind to almost everyone | | | |  | |  | |  | | |  | | |  |
| Does things efficiently | | | |  | |  | |  | | |  | | |  |
| Remains calm in tense situations | | | |  | |  | |  | | |  | | |  |
| Prefers work that is routine | | | |  | |  | |  | | |  | | |  |
| Is outgoing, sociable | | | |  | |  | |  | | |  | | |  |
| Is sometimes rude to others | | | |  | |  | |  | | |  | | |  |
| Makes plans and follows through with them | | | |  | |  | |  | | |  | | |  |
| Gets nervous easily | | | |  | |  | |  | | |  | | |  |
| Likes to reflect, play with ideas | | | |  | |  | |  | | |  | | |  |
| Has few artistic interests | | | |  | |  | |  | | |  | | |  |
| Likes to cooperate with others | | | |  | |  | |  | | |  | | |  |
| Is easily distracted | | | |  | |  | |  | | |  | | |  |
| Is sophisticated in art, music, or literature | | | |  | |  | |  | | |  | | |  |
